# Analyzing the cancer mortality-to-incidence ratios and health expenditures in the aging population: a 20-year comparative study across high-income countries

**DOI:** 10.3389/fragi.2025.1506897

**Published:** 2025-01-22

**Authors:** Majed Ramadan, Shadell AlGhamdi, Rawiah Alsiary

**Affiliations:** ^1^ Population Health Research Section, King Abdullah International Medical Research Center (KAIMRC), King Saud Bin Abdulaziz University for Health Sciences, Ministry of National Guard–Health Affairs, Jeddah, Saudi Arabia; ^2^ College of medicine, King Saud bin Abdulaziz University for Health Science, Ministry of National Guard–Health Affairs, Jeddah, Saudi Arabia; ^3^ Department of Cellular Therapy and Cancer Research, King Abdullah International Medical Research Center (KAIMRC), King Saud Bin Abdulaziz University for Health Sciences, Ministry of National Guard–Health Affairs, Jeddah, Saudi Arabia

**Keywords:** older adults, cancer, human development index, health expenditures, mortality to incidence ratio

## Abstract

**Background:**

The global burden of cancer is expected to increase by 60% over the next two decades, largely due to population aging. The study aims to examine the association between cancer mortality-to-incidence ratios (MIR) with healthcare expenditures (HE), and human development index score for individuals 70 years old or older.

**Method:**

This is an epidemiological study using publicly available data from the Global Burden of Disease (GBD) for six over the years 1990–2019. A generalized linear model was employed to examine the association between MIR, and health expenditures and health development index score.

**Results:**

Included countries showed a statistically significant negative association between MIR and both HE, and HE, indicating that higher HDI and HE are associated with decreased MIR with the highest decrease was for China, the coefficient for HDI is −1.29 (95% CI: –1.35 to −1.24, p < 0.0001), the coefficient for HE is −0.103 (95% CI: –0.17 to −0.03, p < 0.0001). There are variations exist in MIRs between high and low health expenditure countries for each cancer type.

**Conclusion:**

The study reveals a significant impact of HE and HDI on cancer outcomes in older adults. Variations between high and low HE nations highlight potentially improved cancer outcomes in high HE countries. Considering the anticipated growth in the aging population worldwide, a rise in cancer cases is expected among older individuals. The implications are profound, suggesting an impending strain on healthcare systems, particularly in nations with a high proportion of elderly and low health expenditures.

## Introduction

Cancer is a leading global cause of death, and presents escalating incidence and mortality rates, particularly among the elderly (Pilleron et al., 2019). By 2035, the largest relative worldwide increases in all types of cancer incidence among the older population is predicted in the Middle East and Northern Africa (+157%), and in China (+155%) (Pilleron et al., 2019; DeSantis et al., 2019). The global burden of cancer is expected to increase by 60% over the next two decades, largely due to population aging (DeSantis et al., 2019; Siegel et al., 2023). Population aging significantly impacts the overall landscape of cancer-related deaths, with the aging demographic facing an extended risk of developing cancer (DeSantis et al., 2019; Siegel et al., 2023). This elevated risk, notably observed in specific cancers like lung, prostate, and breast cancers, underscores the imperative to understand the evolving dynamics as the population ages (Li et al., 2021; Yeh et al., 2020). The vulnerability of older individuals to the effects of cancer, attributed to a potentially weak ability to combat the disease, contributes to an elevated mortality rate in this age group. Considering this scenario, the global burden of cancer is anticipated to surge by 60% in the next two decades, predominantly due to population aging (Sleeman et al., 2019). This projected increase holds profound implications for governmental healthcare systems and economies globally (Sleeman et al., 2019). As countries worldwide undergo a demographic shift toward aging populations, efforts to prevent and treat cancer must adapt to the challenges posed by population aging (Leeson, 2018; Li et al., 2020). The anticipated rise, ranging from 5% to 30% in aging populations by 2050 (Leeson, 2018; Li et al., 2020; Qiu et al., 2021), reinforces the urgency of addressing chronic diseases, including cancer, in the context of aging.

Conflicting research findings regarding the impact of health expenditures (HE) *per capita* on cancer incidence and mortality rates add complexity to the landscape (Philipson et al., 2012; Soneji and Yang, 2015; Stevens et al., 2015). While some studies suggest a correlation between higher healthcare spending and lower cancer mortality rates, others find no observed difference in outcomes based on healthcare spending patterns. In this context, epidemiologists introduced the Mortality-to-Incidence Ratio (MIR) as a crucial tool for understanding cancer prognosis and shaping effective cancer management and surveillance (Choi et al., 2017; Sharma, 2022; Cordero‐Morales et al., 2016; Vostakolaei et al., 2011; Chow et al., 2022). MIR is an indicator of how well a population does after receiving a cancer diagnosis. High MIR values for a region are an indicator of poor cancer outcomes relative to incidence, thereby indicating areas for targeting interventions related to access to screening, treatment and improved survivorship care (Choi et al., 2017; Sharma, 2022; Cordero‐Morales et al., 2016; Vostakolaei et al., 2011; Chow et al., 2022). Given that nearly half of cancer-related mortality occurs in individuals aged 70 or older (Miller et al., 2016; Huang et al., 2022), our study hypothesizes that HE *per capita* and the human development index score (HDI) influence the cancer MIR in this age group.

The objectives of our study align with these hypotheses. Firstly, we aim to evaluate the dynamic trends in MIR for all cancers over the past two decades, specifically focusing on individuals aged 70 and above. Secondly, our study seeks to examine the association between cancer MIR and healthcare expenditures, along with the human development index score for older adults. This investigation spans a diverse sample of high-income countries characterized by varying levels of overall governmental healthcare spending. The study is motivated by the need to comprehend and address the complex interplay between cancer outcomes, healthcare investments, and socio-economic development in the aging population.

## Methods

### Source of data

We used multiple sources of data to obtain the data utilized in the current study. For epidemiological data on cancer age-specific incidence, and mortality, the data was obtained from the Global Burden of Disease (GBD) 2019 (https://ghdx.healthdata.org/gbd-2019). The GBD provided inclusive and accessible epidemiological data on 369 diseases and injuries, as well as 87 risk factors, for 200 countries and territories from 1990 to 2019 (Wang et al., 2020). All data from the GBD 2019 dataset were included in the analysis without any censoring or removal. Standard data cleaning procedures were applied to ensure the quality and consistency of the dataset, as per the GBD methodological framework. The data for HE *per capita*, were obtained from the World Health Statistics (https://www.who.int/gho/publications/world_health_statistics/en/) of the WHO. The data for HDI was obtained from Our World in Data (https://ourworldindata.org/human-development-index). Our World in Data provides data on global trends across a variety of topics, including health, education, environment, and poverty (Roser et al., 2023). The data used by Our World in Data is gathered from a variety of sources, including the World Bank, the United Nations, the World Health Organization, and other international organizations. The data is collected using a standardized methodology and is regularly updated to ensure accuracy and completeness (Murray et al., 2020).

### Study design, setting, and variables

This epidemiological study utilized publicly available data encompassing six high-income countries with varying HE levels, expressed as a percentage of Gross Domestic Product (GDP), spanning the years 2000–2019. To better comprehend the disparities in the MIRs for different types of cancer across these selected countries, we categorized them into two groups: high health expenditures countries, with expenditures ranging between 7.03% and 12.5% of GDP based on the year 2000 (the United States, Japan, and Germany), and low HE countries, with expenditures between 2.4% and 4.5% of GDP in 2000 (comprising Saudi Arabia, China, and the United Arab Emirates) (United Nations et al., 2021).

Our inclusion criteria encompassed data for both sexes, covering neoplasm incidence and mortality for the 70+ age group, HE as well as HDI scores for these six countries, spanning the period from 2000 to 2019. The MIR was defined as the ratio of the crude mortality rate to the crude incidence rate, as previously described (Sunkara and Hébert, 2015; Chen et al., 2017; Sung et al., 2018). We calculated the Estimated Annual Percentage Change (EAPC) between the values for the year 2000 and those for 2019. Additionally, we conducted a comparative analysis of MIRs across the most prevalent cancers among older adults of both genders in high and low HE countries. These cancers include Breast cancer, Colorectal cancer, Lung cancer, Prostate cancer, Stomach cancer, Liver cancer, Brain cancer, Thyroid cancer, Nasopharynx cancer, Gallbladder and biliary tract cancer, Pancreatic cancer, Ovarian cancer, Kidney cancer, Esophageal cancer, and Larynx cancer (Pilleron et al., 2019; Pilleron et al., 2021).

### Statistical analysis

We presented comprehensive data on age-specific crude incident cases, mortality rates, estimated annual percentage changes (EAPCs), and age-specific MIRs for all cancers, along with 95% uncertainty intervals (UIs). These parameters were essential for quantifying the disease burden within the selected countries. Additionally, we calculated the proportion of age-specific annual incident cases and deaths for all cancer types combined. To assess temporal trends in incidence, mortality, and MIR for all cancers, we estimated the percentage changes in incident cases and deaths, accompanied by EAPCs and their corresponding 95% UIs. The percentage changes in incident cases and deaths for all cancers from 2000 to 2019 were calculated using the equation:

$$\text{Percentage change}=\frac{Incidentcases÷deathcases\;2019-incident\;cases÷death\;cases\;2000\;}{incident\;cases÷death\;cases\;2000}\times 100\%$$

Furthermore, we compiled aggregated average MIRs for each specific cancer type in each of the selected countries to visually illustrate the discrepancies in MIRs between high and low HE countries. To investigate the relationship between MIR and HE, as well as HDI, we employed Generalized Linear Models (GLMs) with 95% confidence intervals. GLMs were chosen due to their flexibility in modeling relationships involving non-normal dependent variables and predictors with varying scales. Appropriate distribution selection was made for each model, considering linearity, homoskedasticity (constant variance), normality, and independence assumptions (Flatt and Jacobs, 2019). This approach allowed for robust analysis of the data’s complex structure. In cases where normality assumptions were violated, the model was adjusted with an appropriate distribution to ensure validity. We reported two-sided p-values with a significant level of 0.05. All statistical analyses were carried out using SAS statistical software version 9.4 (SAS Institute Inc., Cary, NC).

## Results

### Estimated annual percentage change (EAPC) incidence, mortality cure rate and mortality to incidence ratio among adults 70+ from 2000 to 2019

**FIGURE 1:**
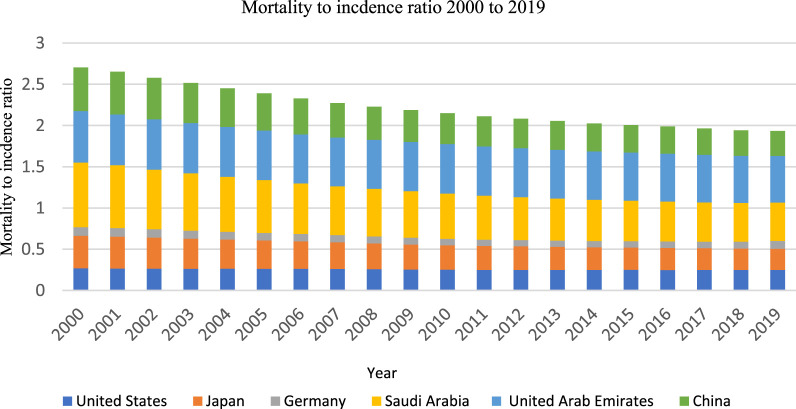
Temporal trends of mortality to incidence ratio.

During the 20-year study period, there was a notable variability in the EAPC for the crude incidence rates of all cancers among the selected countries. The highest increase was observed in the United States, with an EAPC of 32.84% (Uncertainty Interval, UI: 28.19, 35.45). In contrast, Japan experienced the most substantial decrease in EAPC for cancer incidence, with a value of −6.46% (UI: 8.14, −2.02) (Table 1). For crude mortality rates, an overall decline was observed across all selected countries. The United Arab Emirates exhibited the most significant decrease in EAPC, with −30.15% (UI: 27.27, −32.33), while Japan had the smallest reduction, with −2.34% (UI: 1.14, 1.04) (Table 1). Regarding MIR, a decrease in MIR was evident in all countries, except for Germany, where no substantial change was observed between 2000 and 2019. The most remarkable EAPC decrease in MIR was documented in the United States, with a decline of −62.5% (UI: 52.2, −70.2), followed by Japan, which experienced a −50% decrease (UI: 42.81, −55.4) (Table 1; Figure 1).

**TABLE 1:** Incidence crude rate, mortality cure rate and mortality to incidence ratio among adults 70+ from 2000 to 2019.

	Incidence crude populations’ rate (95% UI)^a^			Mortality crude populations’ rate (95% UI)			Mortality-to-incidence ratios (95% UI)		
	2000	2019	Change^b^ (%)	2000	2019	Change^b^ (%)	2000	2019	Change^b^ (%)
Saudi Arabia	11,478.66 (7,953.359, 16,020.26)	11,919.35 (8,327.19, 16,601.24)	3.69 (2.03,5.1)	678.88 (622.44, 734.927)	573.94 (671.92, 491.13)	−18.24 (−16.22, −21.32)	0.06 (0.03, 0.93)	0.05 (0.02, 0.15)	−20 (−16.02, −25.22)
China	6,358.24 (4,589.96, 8,573.74)	6,106.09 (4,605.77, 7,835.56)	−4.12 (−5.09, 1.09)	1,249.19 (1,249.19, 1,337.2)	1,176.11 (1,339.7, 1015.2)	−6.21 (−4.14, 8.19)	0.2 (0.07, 0.53)	0.19 (0.12, 0.42)	−5.26 (−3.11, −7.22)
United Arab Emirates	12,564.57 (17,291.31, 8,728.04)	12,855.31 (8,927.74, 17,856.8)	2.26 (1.01, 3.88)	1,355.2 (1,013.58, 1686.8)	1,041.19 (831.44, 1274.3)	−30.15 (−27.27, −32.22)	0.11 (0.07, 0.45)	0.08 (0.02, 0.12)	−37.5 (−31.21, −40.3)
United States	11,319.82 (9,733.17, 13,068.33)	16,856.45 (14,704.82, 19,092.21)	32.84 (28.19, 35.45)	1,419.15 (1,308.32, 1474.5)	1,293.88 (1,168.6, 1,370.02)	−9.68 (−7.001, −11.08)	0.13 (0.08, 0.32)	0.08 (0.02, 0.13)	−62.5 (−52.2, −70.2)
Japan	13,683.65 (10,225.15, 1,136.972)	12,852.57 (9,933.03, 16,273.66)	−6.46 (−8.14, 2.02)	1,283.32 (1,136.97, 1356.4)	1,253.9 (1,380.3, 1,015.21)	−2.34 (−1.14, 1.04)	0.18 (0.12, 0.22)	0.09 (0.02, 0.16)	−50 (−42.81, −55.4)
Germany	14,829.97 (10,978.28, 19,427.35)	14,684.47 (10,800.94, 19,303.73)	−0.99 (−1.05, 1.09)	1,553.46 (1,421.41, 1626.9)	1,400.22 (1,265.05, 1492.2)	−10.94 (−8.02, 12.06)	0.1 (0.08, 0.17)	0.1 (0.07, 0.16)	—^c^

^a^ UI, uncertainty interval.

^b^ EAPC, estimated annual percentage change.

^c^ No change.

### Health expenditures (HE), and human development index (HDI) score from 2000 to 2019

**FIGURE 2:**
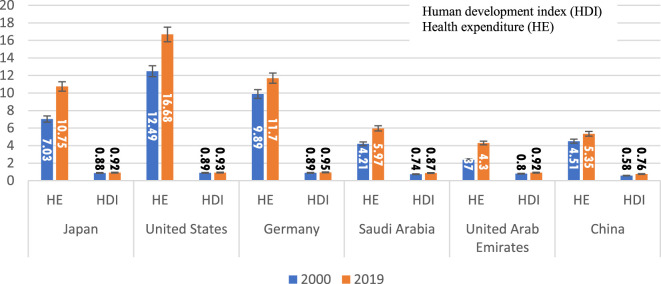
Health expenditure and human development index *per capita* 2000 to 2019.

In 2000, the United States had the highest HE *per capita*, accounting for 12.49% of the Gross Domestic Product (GDP), followed by Germany (9.89%) and Japan (7.03%). Conversely, the United Arab Emirates had the lowest HE *per capita* in 2000, amounting to 2.37% of the GDP (Table 2; Figure 2). HDI scores were relatively close for all countries throughout the study years. The scores ranged from 0.58 in China to 0.89 in the United States in 2000 and from 0.76 in China to 0.95 in Germany in 2019. An incremental and consistent increase in both HE and HDI was observed over the study period for all countries (Table 2; Figure 2).

**TABLE 2:** Health expenditures, and human development index score from 2000 to 2019.

Year	Japan		United States		Germany		Saudi Arabia		United Arab Emirates		China	
	HE^a^	HDI^b^	HE	HDI	HE	HDI	HE	HDI	HE	HDI	HE	HDI
2000	7.03	0.88	12.49	0.89	9.89	0.89	4.21	0.74	2.37	0.8	4.51	0.58
2001	7.24	0.88	13.16	0.89	9.92	0.9	4.46	0.74	2.48	0.8	4.25	0.59
2002	7.35	0.88	13.99	0.89	10.18	0.9	4.25	0.75	2.72	0.8	4.38	0.6
2003	7.49	0.88	14.50	0.9	10.40	0.91	3.98	0.76	2.65	0.81	4.42	0.62
2004	7.54	0.89	14.55	0.9	10.15	0.91	3.58	0.77	2.46	0.82	4.29	0.63
2005	7.66	0.89	14.57	0.9	10.31	0.91	3.42	0.78	2.32	0.82	4.18	0.64
2006	7.69	0.89	14.70	0.9	10.18	0.92	3.62	0.79	2.33	0.83	3.95	0.65
2007	7.78	0.9	14.91	0.91	10.05	0.92	3.56	0.79	2.57	0.83	3.67	0.66
2008	8.09	0.9	15.20	0.91	10.25	0.92	2.97	0.8	2.93	0.83	3.90	0.67
2009	8.96	0.9	16.20	0.91	11.24	0.92	4.29	0.81	4.05	0.83	4.35	0.68
2010	9.06	0.9	16.20	0.91	11.10	0.93	3.65	0.82	3.88	0.84	4.23	0.69
2011	10.49	0.9	16.14	0.91	10.78	0.93	3.71	0.83	3.67	0.84	4.34	0.7
2012	10.67	0.91	16.12	0.92	10.85	0.93	4.02	0.84	3.43	0.85	4.57	0.71
2013	10.67	0.91	15.99	0.92	11.00	0.93	4.47	0.85	3.59	0.85	4.71	0.72
2014	10.73	0.91	16.19	0.92	11.02	0.94	5.23	0.85	3.63	0.86	4.78	0.73
2015	10.75	0.92	16.48	0.92	11.18	0.94	6.00	0.86	3.58	0.87	4.94	0.73
2016	10.66	0.92	16.79	0.92	11.23	0.94	6.43	0.86	3.47	0.87	4.98	0.74
2017	10.66	0.92	16.77	0.92	11.32	0.94	6.26	0.86	4.06	0.90	5.07	0.75
2018	10.75	0.92	16.64	0.93	11.46	0.95	5.54	0.87	4.15	0.91	5.17	0.76
2019	10.75	0.92	16.68	0.93	11.7	0.95	5.97	0.87	4.3	0.92	5.35	0.76

^a^ Health expenditure *per capita* (HE).

^b^ Human Development Index (HID).

### The association between the MIR, and the HE, and HDI *per capita*

**FIGURE 3:**
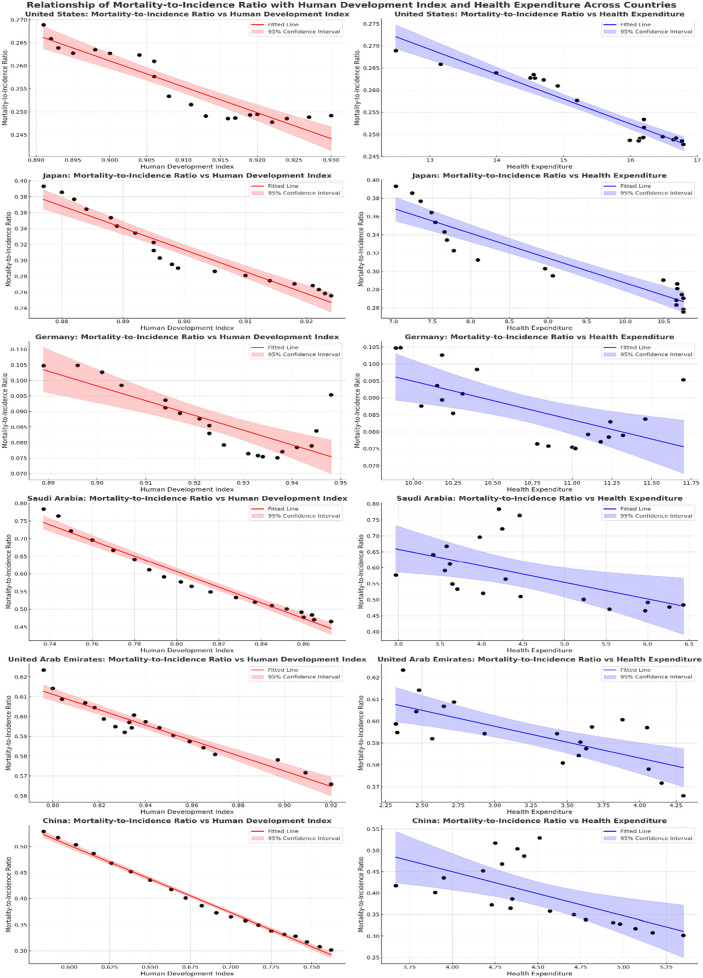
Association between MIR, and HDI, and HE.

Table 3 and Figure 3 display the results of the association between MIR and HDI as well as Health HE for six selected countries. All included countries showed a statistically significant association between MIR and both HE, and HE, indicating that higher HDI and HE are associated with decreased MIR. For China, the coefficient for HDI is −1.29 (95% CI: 1.35 to −1.24, p < 0.0001), the coefficient for HE is −0.103 (95% CI: 0.17 to −0.03, p < 0.0001), in the United Arab Emirates, the coefficient for HDI is −0.39 (95% CI: −0.44 to −0.33, p < 0.0001), and for HE, it is −0.015 (95% CI: −0.02 to −0.007, p = 0.0003), Saudi Arabia shows a HDI (−2.21, 95% CI: −2.42 to −1.99, p < 0.0001) and HE (−0.052, 95% CI: −0.09 to −0.01, p = 0.01), for the United States, the coefficient for HDI is −0.56 (95% CI: −0.67 to −0.45, p < 0.0001), and for HE, it is −0.006 (95% CI: −0.006 to −0.004, p < 0.0001), for Germany HDI (−0.47, 95% CI: −0.65 to −0.29, p < 0.0001) and HE (−0.011, 95% CI: −0.018 to −0.004, p < 0.0001), in Japan, the coefficient for HDI is −2.74 (95% CI: −3.18 to −2.32, p < 0.0001), and for HE, it is −0.027 (95% CI: −0.033 to −0.002, p < 0.0001).

**TABLE 3:** Association between MIR and HDI, and HE.

	Coefficient		Standard error		R-square		95% confidence interval		p-value	
	HDI	HE	HDI	HE	HDI	HE	HDI	HE	HDI	HE
China	−1.29	−0.103	0.02	0.03	0.99	0.39	(−1.35, −1.24)	(−0.17, −0.03)	<0.0001	<0.0001
United Arab Emirates	−0.39	−0.015	0.03	0.003	0.92	0.52	(−0.44, 0.33)	(−0.02, −0.007)	<0.0001	0.0003
Saudi Arabia	−2.21	−0.052	0.01	0.02	0.96	0.29	(−2.42, −1.99)	(−0.09, −0.01)	<0.0001	0.01
United States	−0.56	−0.006	0.05	0.0004	0.87	0.91	(−0.67, −0.45)	(−0.006, −0.004)	<0.0001	<0.0001
Germany	−0.47	−0.011	0.09	0.003	0.62	0.4	(−0.65, −0.29)	(−0.018, −0.004)	<0.0001	<0.0001
Japan	−2.74	−0.027	0.2	0.002	0.91	0.88	(−3.18, −2.32)	(−0.033, −0.002)	<0.0001	<0.0001

Human development index (HDI).

Health expenditures (HE).

### The average MIRs for the common cancer types among older individuals across countries with high and low health expenditures between 2000 and 2019

Table 4 presents the average MIRs for the common cancer types among older individuals across countries with high and low HE *per capita* from 2000 to 2019. There is an overall high MIR’s ratios for those specific type of cancers associated with aging across all countries. There are variations in MIRs between high and low HE countries for each cancer type. In breast cancer MIR is lower in high HE countries (United States 0.32, Japan 0.34, and Germany 0.49) compared to low HE countries (Saudi 0.74 Arabia, China 0.66, and United Arab Emirates 0.74). Similarly, the pattern persists for other cancers.

**TABLE 4:** Average mortality to incidence ratio 2000 to 2019.

	High health expenditures *per capita*			Low health expenditures *per capita*		
	United States	Japan	Germany	Saudi Arabia	China	United Arab Emirates
Breast cancer	0.32	0.34	0.49	0.74	0.66	0.74
Colorectal cancer	0.46	0.49	0.6	0.98	0.76	0.95
Lung cancer	0.88	0.82	0.96	1.19	1.08	1.15
Prostate cancer	0.27	0.37	0.41	0.8	0.68	0.8
Stomach cancer	0.69	0.62	0.84	1.14	1	1.11
Liver cancer	1.03	0.83	0.95	1.13	1.11	1.12
Brain cancer	0.9	0.32	1.08	1.02	1.07	1.06
Thyroid cancer	0.25	0.44	0.58	0.62	0.78	0.64
Nasopharynx cancer	0.64	0.34	0.44	0.96	0.93	1.02
Gallbladder and biliary tract cancer	0.61	0.87	0.79	1.06	1.02	1.03
Pancreatic cancer	1.04	0.94	1.01	1.16	1.14	1.11
Ovarian cancer	0.96	0.84	0.96	1.06	1.05	1
Kidney cancer	0.46	0.72	0.6	0.71	0.79	0.83
Esophageal cancer	1.01	0.73	0.83	1.17	1.09	1.13
Larynx cancer	0.43	0.37	0.71	0.93	0.83	0.95

## Discussion

The current study evaluated the dynamic trends in MIR for all cancers in a 20-year spanning from 2000 to 2019 for older adults across a sample of high-income countries with overall varied aging rate populations and varied governmental healthcare spending. Noteworthy was the great variability in cancer incidence rates, with the United States experiencing the highest surge (32.84%) which might be attributable to the advanced and longtime adoption of screening programs in the U.S. that might have resulted in observing more new cancer cases over the years (Smith et al., 2017). The study also examined the association between cancer MIR and HE, and HDI score. MIR’s witnessed a consistent decrease in all countries, particularly striking in the United States (−62.5%) which might be the result of the high HE *per capita* over the 20-year. Over the 20-year study period, the consistent decrease in MIRs across high-income countries aligns with significant longitudinal improvements in HE and HDI. These changes reflect sustained investments in healthcare infrastructure and socio-economic development, which have supported better cancer care delivery and outcomes. Advancements in treatment, including the adoption of targeted therapies, immunotherapy, and precision medicine, have complemented these trends, particularly in countries with higher healthcare spending. The interplay of these temporal improvements highlights the cumulative impact of economic growth, healthcare investment, and technological progress on cancer outcomes in the aging population. HE and HDI trends revealed the United States leading in *per capita* HE over the study period, while scores for HDI remained relatively close among the included countries, indicating incremental growth. The analysis revealed a significant negative association between increased HE, higher HDI and decreased MIR, emphasizing the role of socio-economic development and healthcare investments reflecting the improvement in older adults’ cancer outcomes. Lastly, the examination of average MIRs for common cancers underscored variations between high and low HE countries, with high-HE nations generally presenting lower MIRs, indicative of potentially improved cancer outcomes. The observed variations in MIRs for specific cancer types likely reflect a combination of factors, including disparities in cancer prevention efforts, access to early detection and treatment, and population-specific risk factors. While these elements were not analyzed in detail in this study, they represent important avenues for future research to better understand and address these differences. Our study reveals that health expenditure has played a role in reducing the MIRs for these specific cancer types in countries with higher health expenditures, such as the U.S., Japan, and Germany. This finding highlights the significant interplay between the outcomes of age-associated cancers and healthcare investments, offering crucial insights for global health policy considerations.

The present findings align with prior research, affirming the significant impact of health expenditures and the human development index on the decrease in MIRs across various cancer types, as indicated in previous studies (Lee et al., 2019; Sung et al., 2021). Notably, our study stands out by specifically focusing on individuals aged +70, a population with an elevated risk of cancer. This emphasis is crucial considering the anticipated growth in the aging population, predicting a surge in cancer cases. The implications are profound, suggesting an impending strain on healthcare systems, particularly in nations with a high prevalence of elderly individuals with cancer. Consequently, the assessment of cancer outcomes in this specific age group becomes imperative for shaping effective cancer management policies and strategies, especially in countries witnessing a notable rise in aging populations. Upon closer examination of common cancer types prevalent in the 70+ age group, there is a consistently elevated MIR across all countries for these specific cancer types. This indicates that the survival outcomes for older adults with age-associated cancers are generally low in the included countries.

In 2019, Japan, the United States, and Germany experienced a significantly higher aging population rate, with a twofold increase compared to China and eight times larger than Saudi Arabia and the United Arab Emirates (Rudnicka et al., 2020). Notably, the sustained substantial investments in healthcare programs and HDI in these countries have played a pivotal role in stabilizing the burden of cancer outcomes. Countries such as Japan, the United States, and Germany have had long-standing healthcare systems, and high HDI investment that have been able to consistently provide high-quality cancer care and treatment, resulting in better outcomes for older cancer patients (Legido-Quigley et al., 2020; Blümel et al., 2020; Miller et al., 2020). On the other hand, low governmental healthcare spending countries have a growing and improving healthcare programs including cancer treatment and prevention that have been reflected on the MIR decrease in older adults’ cancer patients. In addition to the significant advances in cancer treatment in recent years, as new drugs, radiation therapy techniques, and immunotherapies (Debela et al., 2021; Mohan et al., 2019; Pucci et al., 2019). The decline in MIRs observed in this study is not solely attributable to health expenditures and socio-economic development but also reflects significant advances in cancer treatments over the past two decades. Innovations such as targeted therapies, immunotherapy, and precision medicine have revolutionized cancer care, offering more effective and tailored treatments. Additionally, advancements in radiation therapy techniques and the development of supportive care measures have contributed to improved survival rates, particularly in older cancer patients. These technological and therapeutic innovations have likely played a pivotal role in reducing cancer mortality and improving outcomes across high-HE nations. The growing and improving healthcare programs, along with the advances on cancer care, can be attributed to the improvement of cancer outcomes in older adults in the last 10 years of the study period. Overall, our study contributes to the broader understanding of cancer outcomes in the aging population, offering implications for policy development and global healthcare strategies, emphasizing the critical role of sustained healthcare system efficacy in shaping favorable results for older individuals facing cancer challenges.

### Limitations

There are several limitations to our analysis. First, we were only able to estimate the contribution of HR, and HDI to changes in MIR of cancer and did not examine other factors that may have contributed to this increase, such as changes in improvements in cancer treatment or other cancer risk factors. Second, since our reliance on total cancer mortality rates may obscure international variations in cancer type-specific mortality, research is needed to investigate specific types of cancer worldwide. Third, our study’s scope was limited to a sample of high-income countries; thus, the finding cannot be generalized to other populations. Fourth, our reliance on data from the Global Burden of Disease (GBD) 2019 dataset introduces the potential for biases related to data collection and estimation methods. Variations in data quality across countries could influence the results, particularly for countries with less robust health reporting systems. Lastly, the absence of significant confounding factors in the analysis could introduce potential bias, which may weaken the robustness and reliability of the study’s findings.

## Conclusion

The current study concludes that there is a significant impact of HE and HDI on all cancer outcomes among older adults. Upon closer examination of average MIRs for age-associated common cancers, the study indicated that the survival outcomes for older adults with age-associated cancers are generally low in all the included countries. The also study underscored variations between high and low health expenditure countries, with high-expenditure nations generally presenting lower MIRs, indicative of potentially improved cancer outcomes due to continuous high health expenditure. Considering the anticipated growth in the aging population worldwide, a surge in cancer cases is expected among older individuals. The implications are profound, suggesting an impending strain on healthcare systems, particularly in nations with a high proportion of elderly individuals and low health expenditures. This study provides valuable insights into the relationship between HE and cancer MIR in high-income countries. However, future research should consider expanding the analysis to middle- and low-income countries to assess the broader applicability of these findings across different income settings and healthcare systems.

## Data Availability

The original contributions presented in the study are included in the article/supplementary material, further inquiries can be directed to the corresponding author.
